# Source Attribution of Health Burdens From Ambient PM_2.5_, O_3_, and NO_2_ Exposure for Assessment of South Korean National Emission Control Scenarios by 2050

**DOI:** 10.1029/2024GH001042

**Published:** 2024-08-03

**Authors:** Jinkyul Choi, Daven K. Henze, M. Omar Nawaz, Christopher S. Malley

**Affiliations:** ^1^ Environmental Engineering Program University of Colorado Boulder CO USA; ^2^ Department of Mechanical Engineering University of Colorado Boulder CO USA; ^3^ Environmental and Occupational Health Department Milken Institute School of Public Health, George Washington University Washington DC USA; ^4^ Stockholm Environment Institute, Environment Department University of York York UK

**Keywords:** air pollution, health burden, source attribution, emission scenario assessment, GEOS‐chem, adjoint sensitivity

## Abstract

We quantify anthropogenic sources of health burdens associated with ambient air pollution exposure in South Korea and forecast future health burdens using domestic emission control scenarios by 2050 provided by the United Nations Environment Programme (UNEP). Our health burden estimation framework uses GEOS‐Chem simulations, satellite‐derived NO_2_, and ground‐based observations of PM_2.5_, O_3_, and NO_2_. We estimate 19,000, 3,300, and 8,500 premature deaths owing to long‐term exposure to PM_2.5_, O_3_, and NO_2_, respectively, and 23,000 NO_2_‐associated childhood asthma incidences in 2016. Next, we calculate anthropogenic emission contributions to these four health burdens from each species and grid cell using adjoint sensitivity analysis. Domestic sources account for 56%, 38%, 87%, and 88% of marginal emission contributions to the PM_2.5_‐, O_3_‐, and NO_2_‐associated premature deaths and the NO_2_‐associated childhood asthma incidences, respectively. We project health burdens to 2050 using UNEP domestic emission scenarios (Baseline and Mitigation) and population forecasts from Statistics Korea. Because of population aging alone, there are 41,000, 10,000, and 20,000 more premature deaths associated with PM_2.5_, O_3_, and NO_2_ exposure, respectively, and 9,000 fewer childhood asthma incidences associated with NO_2_. The Mitigation scenario doubles the NO_2_‐associated health benefits over the Baseline scenario, preventing 24,000 premature deaths and 13,000 childhood asthma incidences by 2050. It also slightly reduces PM_2.5_‐ and O_3_‐associated premature deaths by 9.9% and 7.0%, unlike the Baseline scenario where these pollutants increase. Furthermore, we examine foreign emission impacts from nine SSP/RCP‐based scenarios, highlighting the need for international cooperation to reduce PM_2.5_ and O_3_ pollution.

## Key Points

- We estimate premature deaths and childhood asthma incidences associated with air pollution exposure in South Korea
- We quantify responses of each health burden to anthropogenic emission changes using adjoint sensitivity analysis
- We project health burdens to 2050 with assessment of domestic emission control scenarios provided by the United Nations Environment Programme

## 1 Introduction

Ambient air pollution is a major health risk factor (Cohen et al., 2017). Exposure to ambient air pollution is associated with negative health outcomes including premature death (Atkinson et al., 2018; Burnett et al., 2014; Huangfu & Atkinson, 2020; Jerrett et al., 2009) and childhood asthma incidences (Achakulwisut et al., 2019). The number of global premature deaths attributable to ambient ground‐level ozone (O_3_) and fine particulate matter (PM_2.5_) exposure was estimated to have increased continuously from 2.3 million in 1990 to 4.5 million in 2019 by the Global Burden of Disease Study 2019 (GBD 2019) (IHME, 2020a; Murray et al., 2020). More recent studies have associated exposure to ambient nitrogen dioxide (NO_2_) with 1.9–4.0 million new pediatric asthma incidences globally in each year (Achakulwisut et al., 2019; Anenberg et al., 2022; Chowdhury et al., 2021) as well as more than 79,000 premature deaths in Europe (Khomenko et al., 2021), 0.25 million premature deaths in China (Xue et al., 2023), and 0.55 million premature deaths in global urban areas (Song et al., 2023).

Reducing ambient air pollution to protect public health is a key target of the United Nations (UN) Sustainable Development Goals. Current ambient air quality standards of the World Health Organization (WHO) are 5 μg m^−3^ annual mean for PM_2.5_, 100 μg m^−3^ 8‐hr mean for O_3_, and 10 μg m^−3^ annual mean for NO_2_ (WHO, 2021). There have been local (C40 Cities, 2019), national (Ministry of Environment of Republic of Korea, 2018; Ministry of Environment, Forest and Climate Change of India, 2019; The State Council of China, 2013; U.S. Environmental Protection Agency, 2011), and regional (European Parliament & Council of the European Union, 2008; UNEP, 2016, 2021) air quality management systems.

Developing effective ambient air quality management systems requires identification of the main sources of air pollution. Air pollution emissions have been studied by using measurements with back trajectory statistics and source‐receptor models (Belis et al., 2014; Hopke, 2016; Oliveri Conti et al., 2017). However, using measurements‐based techniques alone is challenging for estimating contributions of numerous source types and locations to reactive air pollutants. Chemical transport models have been an efficient tool to quantify emission sources of air pollution by either calculating increments based on spatial gradients of concentrations, evaluating impacts of emission perturbations, tagging specific emission sources, or conducting adjoint sensitivity analysis (Thunis et al., 2019). Recently, direct relationships between emissions and air pollution attributable health burdens have been investigated using adjoint sensitivity analysis for cities (Nawaz et al., 2021; Nawaz, Henze, Anenberg, Ahn, et al., 2023), countries (Nawaz & Henze, 2020; Nawaz, Henze, Anenberg, Braun, et al., 2023), regions (Gu, Henze, Nawaz, Cao, & Wagner, 2023; Gu, Henze, Nawaz, & Wagner, 2023), and the globe (Lee et al., 2015). Adjoint sensitivity analysis calculates the linear response of a scalar cost function (e.g., the number of premature death attributable to ambient PM_2.5_ exposure in South Korea) to infinitesimal changes in emissions for each species, sector, and model grid cell. This approach has been widely used to evaluate the health impacts of past emission control policies (Gu, Henze, Nawaz, Cao, & Wagner, 2023; Gu, Henze, Nawaz, & Wagner, 2023; Nawaz et al., 2021; Nawaz & Henze, 2020) as well as to assess the consequences of future emission scenarios (Nawaz, Henze, Anenberg, Ahn, et al., 2023; Nawaz, Henze, Anenberg, Braun, et al., 2023).

For South Korea, previous studies have estimated 10,000–22,000 PM_2.5_‐associated premature deaths (Cohen et al., 2017; Nawaz, Henze, Anenberg, Braun, et al., 2023; Oak et al., 2023), 610–1,600 O_3_‐associated premature deaths (Cohen et al., 2017; Nawaz, Henze, Anenberg, Braun, et al., 2023; Oak et al., 2023), 8,600 NO_2_‐associated premature deaths (Oak et al., 2023), and 15,000–27,000 NO_2_‐associated childhood asthma incidences each year (Achakulwisut et al., 2019; Chowdhury et al., 2021). Nawaz, Henze, Anenberg, Braun, et al. (2023) used the GEOS‐Chem adjoint at a 2° × 2.5° resolution to estimate PM_2.5_‐ and O_3_‐associated premature deaths in the Group of Twenty (G20) countries. To compensate for their coarse model resolution, they downscaled and bias‐corrected their exposure estimates (Lee et al., 2015) using satellite‐derived surface PM_2.5_ concentrations at a 0.1° × 0.1° resolution (Van Donkelaar et al., 2016). Their estimates for South Korea were 13,000 PM_2.5_‐associated premature deaths and 810 O_3_‐associated premature deaths in 2010. Oak et al. (2023) used GEOS‐Chem v13.3.4 at a 0.25° × 0.3125° resolution to focus on South Korea. They used population and mortality from national statistics and exposure‐response model parameters from local epidemiological studies (Byun et al., 2021, 2022; Kim et al., 2021). Their estimates were 10,000, 10,000, and 8,700 premature deaths in 2019 associated with PM_2.5_, O_3_, and NO_2_, respectively. For O_3_‐associated premature death, these two previous studies showed a big difference because Oak et al. (2023) calculated mortality from cardiovascular and respiratory disease following Byun et al. (2021) whereas Nawaz, Henze, Anenberg, Braun, et al. (2023) only accounted for mortality from chronic obstructive pulmonary disorder (COPD) following Turner et al. (2016).

Nawaz, Henze, Anenberg, Braun, et al. (2023) also conducted adjoint sensitivity analysis to attribute their estimated health burdens to regional and sectoral emission sources that were taken from HTAPv2.2 emission inventory (Janssens‐Maenhout et al., 2015). They showed that for South Korea in a base year of 2010, domestic emissions accounted for 16% and 30% of the PM_2.5_‐ and O_3_‐associated premature deaths, respectively. The dominant domestic sectoral contributor was estimated to be transportation for both PM_2.5_‐ and O_3_‐associated premature deaths. Using the adjoint‐based emission contributions, they projected the health burdens to 2040 using the ECLIPSEv5a CLE inventory (IIASA, 2021), additional 50% reduction of transportation emissions, and additional energy emission reduction on pace with net‐zero carbon dioxide (CO_2_) target years (2050 for South Korea). However, the study is limited for PM_2.5_ since they did not consider secondary organic aerosols (SOA). Domestic emission contributions to PM_2.5_‐associated premature deaths might have been underestimated because SOA from domestic emissions are considered to dominate organic aerosol budget especially in South Korea (Nault et al., 2018).

South Korea has put in place actions to control emissions and improve air quality over the past few decades, enacting the Enforcement Decree of the Clean Air Conservation Act in 2007, as amended last by Presidential Decree No 27200 of 31 May 2016, and the Special Act on the Improvement of Air Quality in Seoul Metropolitan Area (SMA) in 2015. Aligning with these efforts, the United Nations Environment Programme (UNEP), working with the municipal government, developed two future emission scenarios by 2050; Baseline and Mitigation scenarios (UNEP, 2023). The Baseline scenario includes only the current policies and measures to improve air quality. On the other hand, the Mitigation scenario reflects on introduction of 11 new policies and measures to achieve carbon neutrality by 2050 and four additional policies and measures to further reduce air pollution.

In this study, we quantify the health impacts of emission changes associated with ambient air pollution in South Korea and provide assessment of the UNEP emission scenarios by 2050. We first estimate PM_2.5_‐, O_3_‐, and NO_2_‐associated premature deaths and NO_2_‐associated childhood asthma incidences using GEOS‐Chem model simulations, satellite‐derived surface NO_2_ concentrations, and ground‐based observations of PM_2.5_, O_3_, and NO_2_ for a base year of 2016. Responses of each health burden to emission changes are calculated using GEOS‐Chem adjoint sensitivities. Next, we apply our adjoint‐based emission contributions to emission reduction ratios of anthropogenic carbon monoxide (CO), nitrogen oxides (NO_x_), non‐methane volatile organic compounds (VOCs), sulfur oxides (SO_x_), ammonia (NH_3_), and PM_2.5_ provided by the UNEP future emission scenarios to project health burdens by 2050 and estimate the corresponding health benefits. Emission reduction ratios for outside of South Korea are taken from nine Shared Socioeconomic Pathway/Representative Concentration Pathway (SSP/RCP)‐based scenarios. Lasty, we discuss uncertainties in our health burden estimation and future projection.

## 2 Materials and Methods

### 2.1 Air Quality Modeling

We simulate PM_2.5_, O_3_, and NO_2_ for South Korea in 2016 using the GEOS‐Chem adjoint model v35, driven by GEOS‐FP meteorology over Northeast Asia [20° × 50°N, 100° × 140°E] at a 0.25° × 0.3125° resolution (Bey et al., 2001). Our model configuration follows the setup described in Choi et al. (2022), incorporating recent advancements pertinent to the 2016 KORUS‐AQ campaign conducted in South Korea (Crawford et al., 2021). The updates include: (a) optimized background methane concentrations (Maasakkers et al., 2019), (b) chemistry updates introduced in the GEOS‐Chem forward model v10, (c) aromatic chemistry (Oak et al., 2019; Porter et al., 2017), (d) C_2_H_4_ chemistry (Kwon et al., 2021), (e) correction of daytime planetary boundary layer height (Oak et al., 2019), and (f) simple anthropogenic SOA formation (Nault et al., 2021). Anthropogenic emissions are from KORUSv5 (Woo et al., 2020), biogenic emissions from MEGANv2.1 (Guenther et al., 2012), and biomass burning emissions from QFEDv2.5 (Koster et al., 2015; Pan et al., 2020). We scale monthly emissions of NO_x_ and CO using the Jet Propulsion Laboratory chemical reanalysis products (Miyazaki et al., 2019). Anthropogenic emissions for the year 2016 are increased by 16% for NO_x_ and 63% for CO.

Low biases of simulated VOCs have been observed in South Korea as shown by a multi‐model intercomparison study during the KORUS‐AQ campaign (Park et al., 2021). Improving the performance of VOC simulations is important for accurate modeling O_3_ (Choi et al., 2022). Therefore, we optimize emissions of total VOCs using a monthly Iterative Finite Difference Mass Balance inversion at a 2° × 2.5° resolution with the Ozone Monitoring Instrument (OMI) CH_2_O vertical column densities as described in Choi et al. (2022). As a result of the CH_2_O inversion, total anthropogenic VOC emissions across the study domain increase by 44%, from 19.6 TgC/year to 28.2 TgC/year. Consequently, anthropogenic emissions of SOA precursors (SOAP), estimated from anthropogenic CO and VOC emissions, are increased by 47% (Nault et al., 2021).

Instead of using the lowest model level O_3_, of which the midpoint altitude is approximately 58 m, we estimate the 2 m O_3_ concentrations for our exposure metric, because this is approximately the average population height (Text S1 in Supporting Information S1). This adjustment accounts for near‐surface concentration reductions due to dry deposition (Zhang et al., 2012). For NO_2_, we apply satellite downscaling and rescaling for NO_2_ (Cooper et al., 2020, 2022; Nawaz et al., 2021). We refine the simulated surface NO_2_ mixing ratios by downscaling using the TROPOspheric Monitoring Instrument (TROPOMI)‐derived surface NO_2_ concentrations in 2019 at a 0.01° × 0.01° resolution. This improves the spatial resolution of our NO_2_ exposure estimates. Additionally, we rescale the NO_2_ concentrations to correct for model biases by using OMI‐derived surface NO_2_ concentrations in 2016 at the model resolution of 0.25° × 0.3125°.

For our health burden calculation described in the following section, we make further adjustments using AirKorea ground observations measured at year‐round sites operated by the Korea National Institute of Environmental Research (airkorea.or.kr). We apply uniform national‐scale adjustment factors to the simulated concentrations based on regression through the origin against the ground observations. For these regressions, the observations are regridded to the 0.01° × 0.01° resolution for NO_2_ and to the model 0.25° × 0.3125° resolution for PM_2.5_ and O_3_. The number of regridded observation points is 85 for the 0.25° × 0.3125° resolution and 322 for the 0.01° × 0.01° resolution. These observation points cover 3% and 89% of the total South Korean population, respectively. The concentration scaling factors are 0.67, 0.81, and 1.15 for PM_2.5_, O_3_, and NO_2_, respectively.

### 2.2 Health Burden Calculation

We calculate the number of premature deaths associated with ambient exposure to PM_2.5_, O_3_, and NO_2_, and the number of NO_2_‐associated childhood asthma incidences. We estimate PM_2.5_‐associated premature deaths due to ischemic heart disease, stroke, chronic obstructive pulmonary disorder (COPD), acute lower respiratory illness, lung cancer, and type‐2 diabetes following the GBD 2019 study (Murray et al., 2020). O_3_‐associated premature deaths are estimated for respiratory diseases including COPD, acute lower respiratory illness, pneumonia, influenza, and other respiratory disease as per Jerrett et al. (2009). For NO_2_‐associated premature death, we follow Atkinson et al. (2018) for lung cancer mortality and Huangfu and Atkinson (2020) for respiratory and cardiovascular disease mortality, including ischemic heart disease. NO_2_‐associated childhood asthma incidences are estimated following Achakulwisut et al. (2019).

First, we calculate the baseline health burdens (B) which can be either premature deaths (D) or childhood asthma incidences (I). The number of premature deaths due to a specific disease d (D_d_) is calculated as

(1) $$D_{d}=MR_{d}\times Pop_{>30yo},$$

where MR_d_ is the baseline mortality rate for disease d and Pop_>30yo_ is the population older than 30 years. Mortality rates and the 2016 population data for South Korea are sourced from Korean National Statistics (kostat.go.kr). Mortality rates are detailed by 10‐year age groups, while population data is broken down by 5‐year age groups and municipal level including cities (Si), counties (Gun), and districts (Gu). We regrid the municipal‐level population data to resolutions of 0.01° × 0.01° and 0.25° × 0.3125° resolutions.

Childhood asthma incidences (I_d = asthma_) are calculated as:

(2) $$I_{asthma}=IR_{asthma}\times Pop_{<15yo},$$

where IR_asthma_ is the asthma incidence rate from the GBD study (IHME, 2020b), and Pop_<15yo_ is the population younger than 15 years. The total estimated childhood asthma incidences in South Korea for 2016 are 68,000.

Next, the health burden attributable to ambient exposure to air pollutant X for health outcome d (B_X,d_) is calculated using an exposure‐response model:

(3) $$B_{X,d}=B_{d}\left(1-\frac{1}{RR_{X,d}}\right),$$

where RR_X,d_ is the relative risk of health burden from ambient exposure to X on d. For O_3_ and NO_2_, RR_X,d_ is calculated using the log‐linear model as:

(4) $$RR_{X,d}=HR_{X,d}^{\frac{\left[X\right]-TMREL_{X}}{\Delta X}},$$

where HR_X,d_ is the hazard ratio associating the health burden (B_X,d_) and ambient exposure to X, provided by epidemiological studies, [X] is the exposure metric, TMREL_X_ is the theoretical minimum risk exposure level, and ΔX is the concentration increment changes to which the health burden is attributable.

For O_3_‐associated premature death, $HR_{O_{3},d}$ is 1.04 (95% uncertainty interval [UI] 1.013–1.067) per ΔO_3_ = 10 ppbv for respiratory disease, with [O_3_] being the warm season average daily maximum 1‐hr O_3_, and $TMREL_{O_{3}}$ is 33.3 ppbv (Jerrett et al., 2009). For NO_2_‐associated premature death, $HR_{NO_{2},d}$ per ΔNO_2_ = 10 μg m^−3^ is sourced from Atkinson et al. (2018) and Huangfu and Atkinson (2020), using annual average NO_2_ as [NO_2_]. $HR_{NO_{2},d}$ is 1.03 (UI 1.00–1.05) for respiratory disease, 1.03 (UI 1.02–1.05) for cardiovascular disease, and 1.05 (UI 1.0–1.08) for lung cancers. The $TMREL_{NO_{2}}$ is set at 4.5 μg m^−3^ (Carey et al., 2013), the reported minimum for most causes of death in Huangfu and Atkinson (2020). In our sensitivity analysis, $D_{NO_{2},d}$ increases by 12% when $TMREL_{NO_{2}}$ is set to 0 μg m^−3^ and decreases by 32% when $TMREL_{NO_{2}}$ is set to 20 μg m^−3^, both compared to the baseline $TMREL_{NO_{2}}$ of 4.5 μg m^−3^. For NO_2_‐associated childhood asthma incidences, $HR_{NO_{2,},asthma}$ is 1.26 (UI 1.10–1.37) per ΔNO_2_ = 10 ppbv, with annual average NO_2_ as [NO_2_], and $TMREL_{NO_{2}}$ is 2 ppbv (Achakulwisut et al., 2019).

For PM_2.5_‐associated premature death $\left(D_{PM_{2.5},d}\right)$, the GBD 2019 study provides look‐up tables that correlate annual average PM_2.5_ concentrations ([PM_2.5_]) with their corresponding relative risks $RR_{PM_{2.5},d}$ and the uncertainties in these relative risks. In the GBD 2019, the $TMREL_{PM_{2.5}}$ was determined from a uniform distribution ranging between 2.4 μg m^−3^ and 5.9 μg m^−3^. In this study, we have chosen not to adopt the TMREL for PM_2.5_ because the estimated minimum of [PM_2.5_] in South Korea (9.9 μg m^−3^) significantly exceeds the $TMREL_{PM_{2.5}}$ range (Section 3.1), and recent studies have identified health impacts associated PM_2.5_ concentrations even below 2 μg m^−3^ (Christidis et al., 2019; The International Council on Clean Transportation, 2023). When we perform a sensitivity analysis using $TMREL_{PM_{2.5}}$ values of 2.4 μg m^−3^ and 5.9 μg m^−3^, the calculated premature deaths due to PM_2.5_ exposure $\left(D_{PM_{2.5},d}\right)$ are reduced by 11% and 26%, respectively.

For uncertainty estimate, we calculate health burdens using the uncertainty interval of HR_X,d_ (or RR_X,d_ for $D_{PM_{2.5},d}$). The uncertainties in health burden estimation are discussed in details in Section 3.5.

### 2.3 Source Attribution

We conduct adjoint sensitivity analysis to calculate the health burdens' responses to precursor emissions from anthropogenic sources (Nawaz, Henze, Anenberg, Braun, et al., 2023). For the adjoint sensitivity analysis, we define four cost functions (J_B,X_) as shown in Equation 5: the number of PM_2.5_‐associated premature deaths $\left(J_{D,PM_{2.5}}\right)$, O_3_‐associated premature deaths $\left(J_{D,O_{3}}\right)$, NO_2_‐associated premature deaths $\left(J_{D,NO_{2}}\right)$, and NO_2_‐associated childhood asthma incidences $\left(J_{I,NO_{2}}\right)$.

(5) $$J_{B,X}=\sum_{d}B_{X,d}$$

The marginal response of each cost function (J_B,X_) to emissions (E_sp,i_) from each anthropogenic species (sp) and model grid cell (i) is calculated as follows:

(6) $$\lambda_{B,X,sp,i}=\nabla_{E_{sp,i}}J_{B,X}=\frac{\partial J_{B,X}}{\partial E_{sp,i}}$$

Here, λ_B,X,sp,i_ is the adjoint sensitivity of J_B,X_ to E_sp,i_, indicating the local‐linear responses of the cost function to incremental emission changes. By the chain rule, $\frac{\partial J_{B,X}}{\partial E_{sp,i}}$ can be expressed combining Equations (3), (4), (5) as:

(7) $$\frac{\partial J_{B,X}}{\partial E_{sp,i}}=\sum_{d}\left(\frac{\partial J_{B,X}}{\partial RR_{X,d}}\cdot \frac{\partial RR_{X,d}}{\partial \left[X\right]}\cdot \frac{\partial \left[X\right]}{\partial E_{sp,i}}\right)$$

It is important to note that nonlinearities are observed in the response of PM_2.5_ (Thunis et al., 2021; Zhao et al., 2019), O_3_ (Cohan et al., 2005; Gu, Henze, Nawaz, & Wagner, 2023; Xing et al., 2011) and NO_2_ (Konovalov et al., 2010) to emissions. This suggests our linear estimates do not fully capture the responses for larger emission changes (greater than 50%) (Nawaz, Henze, Anenberg, Braun, et al., 2023). Consequently, we assume these responses remain valid for up to a 20% reduction in emissions. Thus, we present emission contributions (ΔJ_B,X,sp,i_) as the impacts of a 20% emission reduction (0.2E_sp,i_) on the cost function, as shown in Equation 8.

(8) $$\Delta J_{B,X,sp,i}\approx \lambda_{B,X,sp,i}\times 0.2E_{sp,i}$$

Uncertainties in adjoint‐based emission contributions are further explored in Section 3.3, where we conduct two additional sensitivity simulations; (a) a simulation with boundary conditions set to zero to understand the effects of sources beyond the simulation domain, and (b) a simulation with zero anthropogenic emissions to investigate nonlinearities in the response of health burdens to emissions.

### 2.4 Projection of Future Health Burdens

To estimate future health burdens associated with ambient air pollution exposure, we use population forecasts from Statistics Korea (https://kostat.go.kr/anse/), the emission reduction ratios in future scenarios, and adjoint sensitivities detailed in Section 2.3. We use the population forecasts (Figure S1 in Supporting Information S1) to calculate baseline health burdens in future years (2030, 2040, and 2050) using the 2016 emissions described in Section 2.1. Because of South Korea's low birth rate, the population younger than 15 years old in 2050 is expected to be 39% smaller than that in 2016, whereas the population older than 30 years is 12% larger.

We use ratios of emission reductions forecast in future scenarios for anthropogenic CO, NO_x_, VOCs, SO_x_, NH_3_, and PM_2.5_. For South Korea, we use the Baseline and the Mitigation scenarios provided by the UNEP (2023). The UNEP emission scenarios focus on policies and measures implemented specifically in Seoul, Incheon, and Gyeonggi (SIG). The SIG area, or so‐called SMA, covers 12% of South Korea but contains 50% of the total population and produced 48% of the national Gross Domestic Product in 2016.

**Figure 1:**
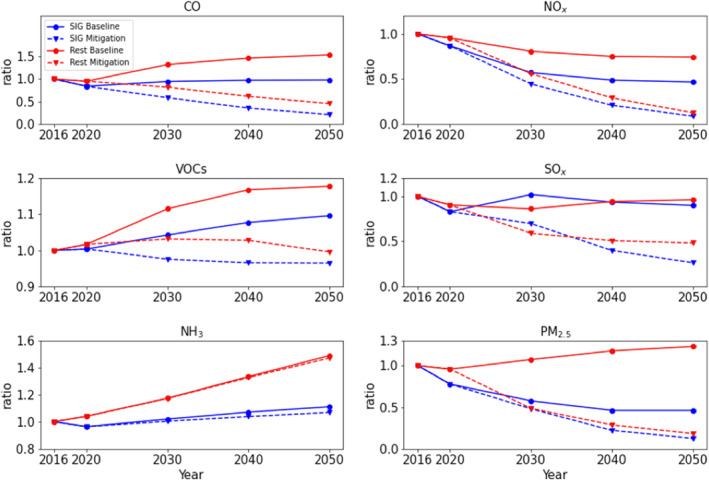
Emission reduction ratios of anthropogenic CO, NO_x_, VOCs, SO_x_, NH_3_, and PM_2.5_ in SIG (red) and in the rest of the country (blue) provided by the United Nations Environment Programme Baseline (solid lines) and Mitigation scenarios (dashed lines).

The Baseline scenario includes current policies and measures to mitigate air quality, such as vehicle emission standards, energy efficiency standards, transitioning to electromobility, and waste reduction (Table S1 in Supporting Information S1). Under the Baseline scenario, NO_x_ emissions are reduced by 53% in SIG and 26% in the rest of the country by 2050 (Figure 1). Other species are not efficiently controlled except for PM_2.5_ emissions in SIG, which is reduced by 54% by 2050. In the rest of the country, emissions are increased for CO, VOCs, NH_3_, and PM_2.5_ by 54%, 18%, 49%, and 23%, respectively. In SIG, emission increases are relatively suppressed but are still significant for VOCs and NH_3_ (by 10% and 11%, respectively).

The Mitigation scenario reflects introduction of new policies and measures to achieve carbon neutrality by 2050 and additional policies and measures to further reduce air pollution (Table S2 in Supporting Information S1). These include zero emission vehicle deployment, transport demand management, fuel economy improvements, non‐road transport mitigation, shipping mitigation, energy efficiency improvement in buildings, a building emissions cap program, renewable electricity and heat generation, an industrial facility emission program, and direct landfill bans. Under the Mitigation scenario, NO_x_ emissions are decreased by 91% in SIG and 87% in the rest of the country by 2050. By 2050, emissions of CO, SO_x_, and PM_2.5_ are also efficiently reduced by 89%, 84%, and 88% in SIG, respectively.

Emissions of NH_3_ are not targeted by future policies or measures. There are little differences in NH_3_ emissions between the Baseline and the Mitigation scenario. By 2050, NH_3_ increase by 11% and 7% in SIG and 49% and 47% in the rest of the country under the Baseline and the Mitigation scenario, respectively. Emissions of VOC are not aggressively targeted, although the Mitigation scenario keeps them from increasing, in contrast to the Baseline scenario. Under the Mitigation scenario, the VOC emission reduction ratio in 2050 is 4% and 0% in SIG and the rest of the country, respectively.

For the rest of the simulation domain outside of South Korea, we use nine SSP/RCP‐based scenarios listed in Table S3 in Supporting Information S1 (Calvin et al., 2017; Fricko et al., 2017; Fujimori et al., 2017; Kriegler et al., 2017; Riahi et al., 2017; van Vuuren et al., 2017) that have been provided for the Coupled Model Intercomparison Project phase 6 (CMIP6) to estimate ranges of foreign emission impacts (Gidden et al., 2019; Rogelj et al., 2018).

Lastly, we assess the health impacts of the future emission scenarios using adjoint sensitivities (λ_B,X,sp,i_). The adjoint sensitivities are applied to the emission reduction ratios in the future scenarios. The adjoint‐based projections are particularly efficient for analysis of multiple scenarios, as no additional forward model calculations are required. Future health burdens (J_B,X,sp,i,t_) in year t are estimated using a first‐order approximation as

(9) $$J_{B,X,sp,i,t}\approx \lambda_{B,X,sp,i}\times \left(1-\gamma_{sp,i,t}\right)E_{sp,i},$$

where γ_sp,i,t_ is the emission reduction ratio of E_sp,i_ in year t. We acknowledge limitations of the approach for large emission changes owing to nonlinearities in the relationship between emissions and health burdens. To investigate the uncertainties in this first‐order approximation, we carry out six additional forward model simulations using the future emissions from the UNEP Baseline and Mitigation scenarios for the years 2030, 2040, and 2050. Nonlinearities in the future health burden response to emission changes are discussed in Section 3.5.

## 3 Results and Discussions

### 3.1 Health Burdens

**Figure 2:**
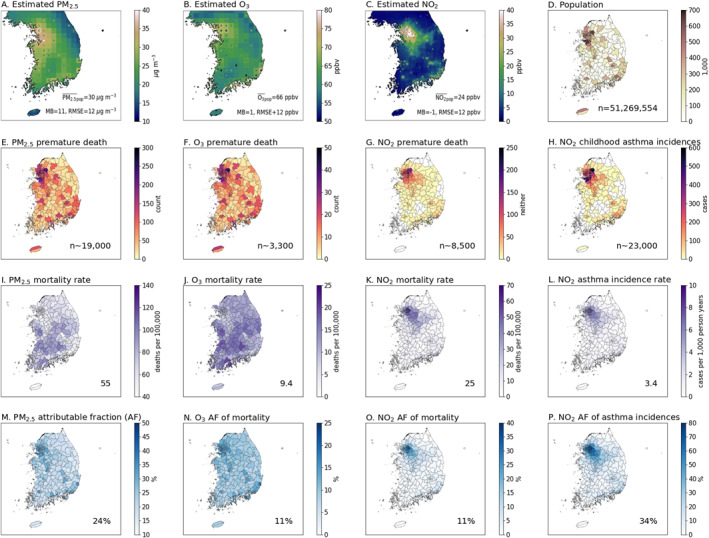
(a–c) Estimated air pollution exposure (background) and ground‐based observations regridded into the corresponding horizontal resolution (circles) are shown for (a) the annual average PM_2.5_ concentrations in μg m^−3^, (b) the warm season average daily maximum 1‐hr O_3_ concentrations in ppbv, and (c) the annual average NO_2_ concentrations in ppbv. The population weighted average of the exposure estimation, and MB and RMSE in comparison with the ground observations are provided in each panel. (d) Population distribution in 2016 by cities (Si‐Gun‐Gu). (e–h) Estimated health burden in count by cities: (e) PM_2.5_‐associated premature death, (f) O_3_‐associated premature death, (g) NO_2_‐associated premature death, and (h) NO_2_‐associated childhood asthma incidences. The total number of health burdens is written in each panel. (i–j) Estimated mortality attributable to (i) PM_2.5_, (j) O_3_, and (k) NO_2_ exposure in deaths per 100,000 population. (l) Estimated childhood asthma incidence rate attributable to NO_2_ exposure in cases per 1,000 person years. (m–o) The fraction of premature mortality attributable to (m) PM_2.5_, (n) O_3_, and (o) NO_2_ exposure in percent. (p) The fraction of childhood asthma incidences attributable to NO_2_ exposure in percent. The national average for (i–p) is written in each panel.

Our estimates of the population weighted concentrations of annual average PM_2.5_, warm season average daily maximum 1‐hr O_3_, and annual average NO_2_ are 30 μg m^−3^, 66 ppbv, and 24 ppbv, respectively, in 2016 (Figures 2a–2c). Because our concentration estimates are scaled up using ground‐based observations (as described in Section 2.1), the mean biases of our estimated concentrations are 11 μg m^−3^, 1 ppbv, and 1 ppbv for PM_2.5_, O_3_, and NO_2_, respectively.

We estimate 19,000 (95% CI: 13,000–24,000) PM_2.5_‐associated premature deaths, 3,300 (1,100–5,200) O_3_‐associated premature deaths, 8,500 (3,200–13,000) NO_2_‐associated premature deaths, and 23,000 (12,000–28,000) NO_2_‐associated new childhood asthma incidences in South Korea in 2016 (Figures 2e–2h). Here, we point out that the premature deaths associated with PM_2.5_, O_3_, and NO_2_ are estimated separately, and we refrain from adding them together in order to avoid double counting. Approximately 46%, 45%, 75%, and 74% of the PM_2.5_‐, O_3_‐, and NO_2_‐ premature deaths and the NO_2_‐associated childhood asthma incidences occur in SIG, respectively. Significant NO_2_‐associated health burdens occur in SIG considering that SIG contains only 50% of the total population of South Korea.

The national average mortality rate due to ambient PM_2.5_, O_3_, and NO_2_ exposure is estimated to be 55, 9.4, and 25 deaths per 100,000 population (Figures 2i–2k). The mortality rate due to ambient PM_2.5_ and O_3_ exposure is high in rural areas whereas NO_2_‐associated mortality rate is high in SIG. The NO_2_ exposure metric peaks in SIG but has low values in the rest of the country—so low that they rarely exceed $TMREL_{NO_{2}}$. Therefore, the spatial distribution of the NO_2_‐associated mortality depends mostly on the spatial distribution of the NO_2_ exposure. On the other hand, the PM_2.5_ and O_3_ metrics have small spatial variations because of their longer lifetimes compared to NO_2_. As a result, the PM_2.5_ and O_3_ metrics consistently surpass TMREL thresholds for these pollutants nationwide, indicating that there are no regions in South Korea considered safe from health risks due to PM_2.5_ and O_3_ exposure.

Figures 2m–2o illustrates the percentage of health burdens caused by exposure to ambient air pollution, emphasizing the critical link between ambient air pollution and public health. Nearly a quarter (24%) of premature deaths from major health conditions such as ischemic heart disease, stroke, chronic obstructive pulmonary disorder (COPD), acute lower respiratory illness, lung cancer, and type‐2 diabetes are attributable to ambient PM_2.5_ exposure. Exposure to ambient O_3_ is responsible for 11% of premature deaths related to respiratory diseases. Similarly, exposure to ambient NO_2_ accounts for 11% of premature deaths related to respiratory and cardiovascular diseases, including lung cancer. This underscores the urgent need for action to reduce air pollution levels.

Particularly concerning is the effect of NO_2_ on children's health, with the national average incidence rate of NO_2_‐associated childhood asthma at 3.4 cases per 1,000 person‐years (Figure 2l). In areas with elevated NO_2_ levels, such as SIG, this rate increases significantly to 8.8 cases per 1,000 person‐years. On average, 34% of new childhood asthma incidences are attributable to NO_2_ exposure, a figure that rises to 52% in the most affected regions like SIG, as shown in Figure 2p.

### 3.2 Adjoint‐Based Emission Contributions

**Figure 3:**
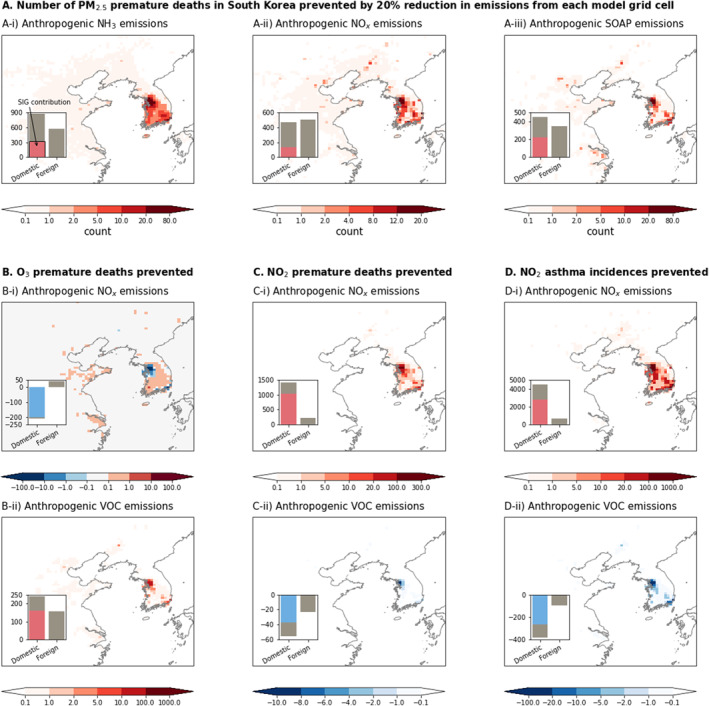
(a) The number of PM_2.5_‐associated premature deaths that can be prevented by a 20% reduction of anthropogenic emissions of (A‐i) NH_3_, (A‐ii) NO_x_, and (A‐iii) SOA precursors. (b) The number of O_3_‐associated premature deaths that can be prevented by a 20% reduction of anthropogenic emissions of (B‐i) NO_x_ and (B‐ii) VOC. (c) The number of NO_2_‐associated premature deaths that can be prevented by a 20% reduction of anthropogenic emissions of (C‐i) NO_x_ and (C‐ii) VOC. (d) The number of NO_2_‐associated childhood asthma incidences that can be prevented by a 20% reduction of anthropogenic emissions of (D‐i) NO_x_ and (D‐ii) VOC. Bar graphs in each panel show the total health benefits for 20% reduction of domestic anthropogenic emissions and foreign anthropogenic emissions. The color bar in each domestic emission contribution shows the proportion of SIG emission contribution.

Here, we focus on the contribution of anthropogenic emissions to each health burden, measured as the number of preventable premature deaths or the number of preventable childhood asthma incidences resulting from a 20% reduction in emissions (Figure 3). Domestic anthropogenic emission contributions to health burdens are 56% for PM_2.5_‐associated premature deaths, 38% for O_3_‐associated premature deaths, 87% for NO_2_‐associated premature deaths, and 88% for NO_2_‐associated childhood asthma incidences. For PM_2.5_‐associated premature deaths, Nawaz, Henze, Anenberg, Braun et al. (2023) reported a much lower contribution (15%) of domestic emissions for the year 2010. This discrepancy is possibly because Nawaz, Henze, Anenberg, Braun, et al. (2023) did not include SOA, which is a significant component of PM_2.5_, especially in South Korea. In this study, we include simple SOA formation from SOAP, whose emissions are increased by 47% by optimizing VOC emissions (Section 2.1). Other factors contributing to the differences between the two studies include the use of different anthropogenic emission inventories (KORUSv5 vs. HTAPv2.2), model spatial resolution (0.25° × 0.3125°vs. 2° × 2.5°), and target years (2016 vs. 2010), which entail different synoptic meteorological conditions (Choi et al., 2019). While uncertainties exist in emission source attribution, significant influences of non‐local sources on PM_2.5_ in South Korea have been noted by various studies (e.g., Tessum et al., 2022), indicating the benefits of collaborative efforts to improve PM_2.5_ air quality.

The top three emitted species that cause PM_2.5_‐associated premature death are NH_3_, NO_x_, and SOAP. We estimate that reducing domestic anthropogenic emissions of NH_3_, NO_x_, and SOAP by 20% could prevent 880, 470, and 450 PM_2.5_‐associated premature deaths in 2016, respectively. Regarding other emission species, a 20% reduction in domestic anthropogenic OC emissions is attributed to preventing 133 PM_2.5_‐associated premature deaths. Reductions of domestic anthropogenic emissions of VOCs, BC, SO_x_, and CO are associated with preventing 73, 42, 26, and zero premature deaths, respectively. For emissions of SO_x_ and CO, contributions of foreign emissions are estimated to be more significant, leading to 110 and 4 PM_2.5_‐associated premature deaths, respectively.

For O_3_‐, and NO_2_‐associated premature deaths and NO_2_‐associated childhood asthma incidences, NO_x_ and VOC emissions account for more than 92%, 99%, and 99% of the emission contributions, respectively. Because of the high NO_x_ concentrations in SIG, the O_3_ concentrations have negative sensitivities to NO_x_ emissions in SIG (Colombi et al., 2023). This condition in SIG significantly influenced our estimated emission contributions to the O_3_‐associated premature deaths, with 95% of the domestic NO_x_ emission contributions originating from SIG (as shown in the bar graph in Figure 3d). If domestic NO_x_ and VOC emissions were reduced by 20%, O_3_‐associated premature deaths would have been increased by 210 and decreased by 240, respectively. In the current high‐NO_x_, VOC‐limited regime of SIG, reducing VOC emissions is the only efficient method to control O_3_ concentrations. However, with significant reductions in NO_x_ emissions, such as 91% decrease projected for SIG by 2050 under the UNEP Mitigation scenario, anthropogenic NO_x_ emissions will become the dominant source, necessitating a reevaluation of emission contributions in this new environment, as suggested by Gu, Henze, Nawaz, and Wagner (2023).

Reducing domestic NO_x_ emissions by 20% could prevent 1,400 NO_2_‐associated premature deaths and 4,500 NO_2_‐associated childhood asthma incidences. On the other hand, VOC emissions have a small, yet negative impact on NO_2_‐associated health burdens because the reaction of NO_2_ with VOC produces organic nitrates, which act as a sink for NO_2_.

### 3.3 Uncertainties in Adjoint‐Based Emission Contributions

In this section, we discuss the uncertainties in adjoint‐based emission source apportionment. Our analysis estimates the total emission contribution to PM_2.5_‐, O_3_‐, and NO_2_‐associated premature deaths, and NO_2_‐associated childhood asthma incidences to be 104%, 67%, 72%, and 91%, respectively. It's important to note that the adjoint‐based total emission contribution does not necessarily sum to 100%. These discrepancies arise because our adjoint‐based calculations rely on tangent linear sensitivities, which explains our decision to focus on 20% emission contributions rather than 100% in Section 3.2. Additionally, our adjoint calculations do not account for sensitivities with respect to boundary conditions; they are limited to sensitivities with respect to emissions within the nested model domain.

To further investigate the uncertainties related to these factors, we conduct two additional sensitivity simulations. In our first sensitivity analysis, we set the boundary conditions to zero to understand the impacts of sources outside of the simulation domain. This results in reduced PM_2.5_‐ and O_3_‐associated premature deaths by 3% and 17%, respectively, quantifying the long‐range transport of PM_2.5_ and O_3_. In contrast, setting zero boundary conditions increases NO_2_ concentrations, and associated premature deaths and childhood asthma incidences increase by 12% and 7%, respectively. This seemingly counter‐intuitive response occurs because the simulation with zero boundary conditions leads to lower levels of imported O_3_. Local NO_2_ concentrations have negative sensitivities to local O_3_, as a reduction in O_3_ decreases nitrate formation, which acts as a sink for NO_2_.

In the second sensitivity analysis, we set anthropogenic emissions within the simulation domain to zero. This zero‐out anthropogenic emission simulation shows reductions of health burdens attributable to PM_2.5_ and O_3_ of 76% and 91%, respectively. By comparison, the adjoint‐based total anthropogenic emission contribution is 99% (out of 104%) and 40% (out of 67%) to PM_2.5_‐ and O_3_‐associated premature death, respectively. The large discrepancies between the zero‐out and adjoint‐based total anthropogenic contributions indicate strong nonlinearities in the response of PM_2.5_‐ and O_3_‐associated premature death to emissions. Emissions from natural sources become more important for PM_2.5_‐associated premature deaths when anthropogenic emissions are removed. For O_3_‐associated premature death, the anthropogenic emission contribution may be larger when the total emission amount is considered than when incremental emission changes are introduced.

On the other hand, the NO_2_‐attributable health burden was reduced to zero in our zero‐out simulation, which shows that anthropogenic emissions within the simulation domain can explain most of the NO_2_‐attributable health burden. The adjoint‐based emission contribution is also mostly from anthropogenic sources; 73% (out of 72%, reflecting negative sensitivities to biogenic VOC emissions) and 91% (out of 91%) for NO_2_‐attributable premature death and childhood asthma incidences, respectively. In these NO_2_ cases, we adjust the adjoint‐based total emission contribution to 100% of the corresponding NO_2_‐associated health burden: figures in Section 3.2 are based on the adjusted emission contributions. This adjustment is made because we believe the discrepancies in the total emission contributions are due to significant sensitivities of NO_2_ to boundary conditions and local O_3_ concentrations, as partially investigated in the first sensitivity analysis with zero boundary conditions. We assume the sensitivities of NO_2_‐associated health burdens to boundary conditions are negligible when NO_x_ emissions are significantly reduced: NO_x_ emissions in SIG are already reduced by 40% in 2030 under the Baseline scenario and further by 91% in 2050 under the Mitigation scenario.

In contrast, we do not apply this adjustment for O_3_‐associated premature deaths. This is because we recognize nonlinearities as being most significant in the relationship between O_3_ and precursor emissions, whereas we consider the relationship between NO_2_ and precursor emissions to be more linear. We avoid introducing artificial corrections to the highly nonlinear O_3_ system.

### 3.4 Future Projection of Health Burdens

**Figure 4:**
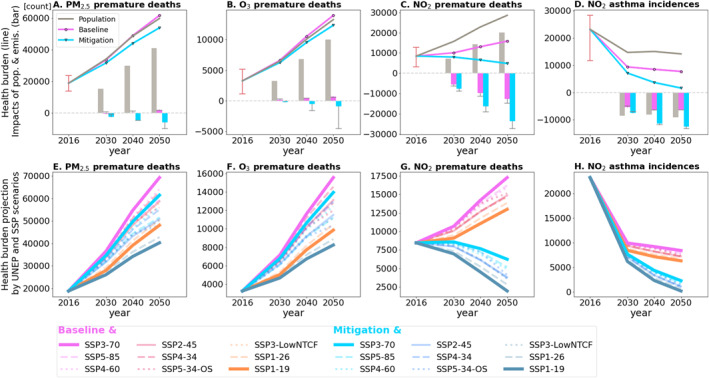
(a–d) Estimated future premature deaths (a–c) and childhood asthma incidences (d) attributable to PM_2.5_ (a), O_3_ (b), and NO_2_ (c, d) exposure projected using dynamic population forecast (gray line) by Statistics Korea and the Baseline (magenta line with circles) and the Mitigation emission scenarios (cyan line with downward triangles) provided by UNEP (2023) and impacts of population (gray bar), the Baseline emissions (magenta bar), and the Mitigation emissions (cyan bar). The red error bar in 2016 shows uncertainties in HR_X,d_. Gray error bars in bar graphs in 2030, 2040, and 2050 show uncertainties in adjoint‐based emission contribution. (e–h). Same as line graphs in panels (a–d) but future foreign emission changes are also included using nine SSP/RCP emission scenarios; SSP1/RCP1.9, SSP1/RCP2.6, SSP2/RCP4.5, SSP3/RCP7.0, SSP3/LowNTCF, SSP4/RCP3.4, SSP4/RCP6.0, SSP5/RCP3.4‐OS, and SSP5/RCP8.5. South Korean emissions follow either the Baseline scenario (magenta lines) or the Mitigation scenario (cyan lines). See Table S4–S8 in Supporting Information S1 for the corresponding data.

We present our estimated future health burdens attributable to air pollution exposure in Figure 4. Because of the population aging in South Korea, the number of premature deaths increases in 2050, despite the total population reducing. Without emission changes, the number of PM_2.5_‐, O_3_‐, and NO_2_‐ attributable premature death increases from 19,000 to 60,000, from 3,300 to 13,000, and from 8,500 to 29,000, respectively, by 2050. On the other hand, the number of NO_2_‐attributable childhood asthma incidences decrease from 23,000 in 2016 to 14,000 in 2050.

The impacts of domestic emission controls are large for the NO_2_‐attributable health burden under both the Baseline and Mitigation scenarios. The health benefits from emission controls under the Baseline scenario are 13,000 (44%) fewer premature deaths and 6,400 (45%) fewer childhood asthma incidences attributable to NO_2_ in 2050. Under the Mitigation scenario, the health benefits are 24,000 (83%) premature deaths and 13,000 (88%) childhood asthma incidences attributable to NO_2_ in 2050.

The health benefits from emission controls are comparably small in terms of PM_2.5_‐ and O_3_‐attributable premature deaths. This is because NH_3_ and VOC emissions are not efficiently targeted by the future scenarios. Under the Baseline scenario, the concentrations of PM_2.5_ and O_3_ even increase, leading to 1,900 (3.2%) and 690 (5.2%) more premature deaths in 2050. Owing to ambitious controls of NO_x_ emissions, the Mitigation scenario results in 5,900 (9.9%) and 920 (7.0%) fewer premature deaths in 2050.

Inclusion of foreign emission changes using nine SSP/RCP scenarios shows a wide range of possible future pathways. The number of PM_2.5_‐attributable premature deaths varies from 40,000 to 69,000 in 2050. The number of O_3_‐attribuatble premature deaths in 2050 can be from 8,200 to 16,000. The NO_2_‐attributable health burden in 2050 is estimated to be from 2,000 to 17,000 premature deaths and 230 to 8,400 childhood asthma incidences.

The impacts of foreign emissions are significant for the PM_2.5_‐ and O_3_‐attributable health burden, for which the ranges in 2050 are 21,000 and 5,700 premature deaths. For the NO_2_‐attributable health burden, the foreign emission impacts are comparably small because the NO_2_ concentrations are mostly from domestic emissions, as discussed in Section 3.2. The ranges of the foreign emission impacts are 4,300 for the NO_2_‐attributable premature death and 2,100 for the NO_2_‐attributable childhood asthma incidences. Direct comparisons between the impacts of domestic and foreign emission controls are not addressed in this study. While domestic emissions follow the two UNEP scenarios that are likely to happen in the future, foreign emissions follow nine SSP/RCP scenarios including the greenest pathway (IMAGE SSP1/RCP1.9) and the most aggressive fossil‐fueled development pathway (REMIND‐MAGPIE SSP5/RCP8.5).

### 3.5 Uncertainties in Estimated Health Burdens

Here, we address the uncertainties in estimating current and future health burdens. We identify four important categories of uncertainties in health burden estimation: (a) uncertainties in air quality estimation, (b) uncertainties in epidemiological concentration‐response functions, (c) uncertainties introduced through application of local‐linear sensitivities for projecting responses of a nonlinear system, and (d) uncertainties in future projection due to meteorological variability.

Uncertainties in air quality estimation are partly addressed in Section 3.1. Figures 2a–2c present comparisons of our estimated air pollution exposure metrics against the AirKorea ground observations. By adjusting our estimates using regression slopes (Section 2.1), we obtain a normalized mean bias (NMB) of only 1%, 0%, and −3% for annual average PM_2.5_, warm season average daily maximum 1‐hr O_3_, and annual average NO_2_, respectively. However, evaluation of the spatial distribution indicates an overestimation in the SIG urban areas: 9% for PM_2.5_, 3% for O_3_, and 18% for NO_2_. The 18% overestimation in NO_2_ in SIG leads to an overestimation of NO_2_‐associated premature deaths and childhood asthma incidences in South Korea by approximately 12% and 10%, respectively, and in SIG by 17% and 13%.

Uncertainties in epidemiological concentration‐response functions stem from uncertainties in each parameter within these functions. Uncertainties in HR_X,d_ lead to 26%, 66%, 62%, and 50% uncertainties in estimating PM_2.5_‐, O_3_‐, and NO_2_‐associated premature deaths, and NO_2_‐associated childhood asthma incidences, respectively (Figures 4a–4d). Beyond HR_X,d_, the choice of exposure metric and TMREL_X_ plays a critical role in estimating health burdens (Section 2.2). Local epidemiological data usage could refine local health burden estimates, as suggested by Oak et al. (2023). For instance, there is a large disparity in O_3_‐associated premature deaths in South Korea: 810 by Nawaz, Henze, Anenberg, Ahn, et al. (2023), Nawaz, Henze, Anenberg, Braun, et al. (2023) versus 10,000 by Oak et al. (2023). This is mostly because Oak et al. (2023) followed Byun et al. (2021), considering cardiovascular and respiratory disease mortality, while Nawaz, Henze, Anenberg, Ahn, et al. (2023), Nawaz, Henze, Anenberg, Braun, et al. (2023) focused on COPD mortality based on Turner et al. (2016), used by GBD 2019. In this study, we reference Jerrett et al. (2009) for the relationship between O_3_ exposure and respiratory disease mortality. Therefore, our estimate of 3,300 premature deaths sits between the figures reported by Nawaz, Henze, Anenberg, Ahn, et al. (2023) and Oak et al. (2023). Other sources of uncertainty include population data, mortality rates, and asthma incidence rate. However, these are likely smaller compared to the aforementioned sources (Ostro et al., 2018). This is partly because we use local population and health data, while applying globally determined concentration‐response functions.

Nonlinearities in the health burden response to emission changes represent a significant source of uncertainty in future projections of health burdens. We find that nonlinearity leads to underestimation of the health benefits of the Mitigation scenario by up to 57%, 81%, 15%, and 18% for PM_2.5_‐, O_3_‐, and NO_2_‐associated premature deaths, and NO_2_‐associated childhood asthma incidences, respectively (Figures 4a–4d). The underestimation is particularly significant for O_3_‐associated premature deaths. As discussed in Section 3.2, the O_3_ concentrations in 2016 are under high‐NO_x_ and VOC‐limited regime, leading to negative sensitivities of the O_3_ health burden to local NO_x_ emissions in SIG. Therefore, applying the adjoint‐based emission contributions calculated in 2016 to future years results in projected negative health benefits from NO_x_ emission reductions in SIG. Conducting additional adjoint sensitivity analysis with future emissions or calculating second‐order emission contributions could help mitigate these uncertainties (Nawaz, Henze, Anenberg, Braun, et al., 2023).

The health benefit underestimation for the PM_2.5_‐associated premature death is only shown in 2050 under the Mitigation scenario when NO_x_ and SO_x_ emissions decreases are substantial (Figure 1). This results from the nonlinear response of inorganic aerosols to precursor emissions. Inorganic aerosols including ammonium $\left(NH_{4}^{+}\right)$, nitrate $\left(NO_{3}^{-}\right)$, and sulfate $\left(SO_{4}^{2-}\right)$ constitute 48% of the population weighted average of PM_2.5_ in 2016 of 30 μg m^−3^. Under the Mitigation scenario in 2050, the population weighted average of PM_2.5_ decreases to 21 μg m^−3^ and 99% of the PM_2.5_ decrease is due to inorganic aerosols. However, adjoint‐based contributions calculated in Section 3.2 are only 24%, 13%, and 1% from domestic anthropogenic emissions of NH_3_, NO_x_, and SO_x_, respectively (not shown). In addition, NH_3_ emissions increase from 2016 to 2050 by 7% in SIG and 47% in the rest of the country. This indicates that NH_3_ is saturated and is no longer a limiting factor controlling PM_2.5_ formation when both NO_x_ and SO_x_ emissions significantly decrease by 2050 under the Mitigation scenario; thus, increases in NH_3_ emissions do not increase PM_2.5_. Also, the impacts of NO_x_ and SO_x_ emissions on PM_2.5_‐associated premature death are larger than the adjoint‐based contributions when emission changes are large.

Lastly, future projections based on the analysis of a single year, such as 2016, are subject to uncertainties due to variations in emission sources influenced by synoptic meteorological conditions (Choi et al., 2019; Nawaz & Henze, 2020). The year 2016 recorded the second‐highest annual mean temperature of 13.6°C since 1973, as reported by Korea Meteorological Administration (2017), attributed to an unusually intense heat wave in August 2016 (Yeh et al., 2018). It is noteworthy that this record was surpassed in 2023, with an annual mean temperature of 13.7°C. The anomalous temperature may have influenced local and transboundary emission patterns and subsequent impacts on air quality. Precipitation‐wise, the precipitation in 2016 amounted to 97.4% of the typical year's precipitation. The uncertainties stemming from meteorological variability could be addressed through additional forward model simulations utilizing future climate prediction models; however, this is a consideration not tackled in this study.

## 4 Conclusion

In this study, we calculate the responses of health burdens attributable to air pollution exposure to changes in anthropogenic emissions and assess the health benefits of the UNEP domestic emission scenarios for South Korea by 2050. Our health burden estimation framework utilizes air quality modeling and exposure‐response modeling. For air quality modeling, we use GEOS‐Chem, satellite‐derived surface concentrations of NO_2_, and year‐round ground‐based observations of PM_2.5_, O_3_, and NO_2_. For exposure‐response modeling, we reference epidemiological studies and use statistical information including population, mortality rates, and asthma incidence rates. We estimate air pollution attributable health burdens for a base year of 2016, including 19,000 PM_2.5_‐associated premature death, 3,300 O_3_‐associated premature death, 8,500 NO_2_‐associated premature death, and 23,000 NO_2_‐associated childhood asthma incidences. We find high attributable fraction (AF) of health burdens to ambient air pollution in South Korea, particularly for NO_2_‐associated childhood asthma incidences, with the national average AF of 34% and a maximum of 52% in SIG. The substantial association between air pollution and health burdens demonstrates the critical need for comprehensive strategies to mitigate air pollution for public health.

Next, we attribute the anthropogenic sources of each health burden in 2016 using adjoint sensitivity calculations. For PM_2.5_‐associated premature death, domestic anthropogenic NH_3_, NO_x_, and SOAP are estimated to be the top‐3 contributors; a 20% emission reduction could lead to 880, 470, and 450 fewer premature deaths, respectively. Responses of O_3_‐associated premature deaths to emission changes are nonlinear. A domestic anthropogenic emission reduction of NO_x_ and VOCs by 20% results in 210 more and 240 fewer premature death, respectively. Contributions of NO_x_ emissions to NO_2_‐associated premature deaths and childhood asthma incidences are more linear. Reducing domestic anthropogenic NO_x_ emissions by 20% could prevent 1,400 and 4,500 NO_2_‐associated premature deaths and childhood asthma incidences.

Lastly, we predict health burdens in 2050 by combining estimated health burdens in 2016, responses of each health burden to emission changes, population forecast, and future emission scenarios (the UNEP Baseline and Mitigation scenarios). The impacts of emission changes cause PM_2.5_ and O_3_‐associated premature deaths to still increase under the Baseline scenario, where NH_3_ and VOC emissions are not controlled. The Mitigation scenario does not efficiently target NH_3_ and VOC emissions either. However, under the Mitigation scenario, we estimate positive yet small health benefits for PM_2.5_‐ and O_3_‐associated premature deaths thanks to ambitious reduction in NO_x_ emissions. For NO_2_‐associated health burdens, both scenarios are estimated to have positive health outcomes. The inclusion of dynamic population forecasts leads to increases in baseline premature deaths and decreases in baseline childhood asthma incidences due to population aging. The health benefits of the Mitigation scenario are shown to be significant for NO_2_‐associated premature deaths; the future health burden increases under the Baseline scenario but decreases under the Mitigation scenario. Additionally, we include foreign emission changes following nine SSP/RCP‐based scenarios and provide a wide range of possible future pathways.

We discuss uncertainties in our estimated future health burdens. Major sources of uncertainty include air quality simulations, exposure‐response modeling, and adjoint‐based emission contribution calculation. We try to minimize uncertainties in air quality simulations by using satellite‐derived NO_2_ and surface observations of PM_2.5_, O_3_ and NO_2_. However, we still estimate uncertainties in the spatial distribution of air pollution, which could result in up to a 20% overestimation of the health burden. For exposure‐response modeling, large uncertainties exist in HR_X,d_ from epidemiological studies, estimated to be between 26% and 66%. There are uncertainties in adjoint‐based emission contributions because adjoint sensitivities are first‐order approximations of the nonlinear responses of health burdens to emission changes. These uncertainties are significant when nonlinearities are strong and emission changes are large; for example, the health benefits of emission changes for O_3_‐associated premature deaths are underestimated by 81% under the Mitigation scenario in 2050 when domestic anthropogenic NO_x_ emissions are decreased by about 90%.

Even given the uncertainties and limitations discussed above, we still identify several robust conclusions of our assessment as follows:

1. Significant associations between health burdens and ambient air pollution exposure in South Korea are identified. The AF is significant especially for NO_2_‐associated childhood asthma incidences. Approximately 32% of new childhood asthma incidences in South Korea are attributable to ambient NO_2_ exposure.
2. NO_2_‐associated health burdens can be addressed domestically. We estimate substantial health benefits of the two UNEP domestic emission scenarios.
3. PM_2.5_‐associated premature deaths can not be addressed by the UNEP scenarios. We forecast more premature deaths under the Baseline scenario because of increases in NH_3_ emissions. We estimate small health benefits of the Mitigation scenario thanks to ambitious NO_x_ emission controls. We demonstrate the need for NH_3_ emission controls for mitigating ambient PM_2.5_.
4. The UNEP Mitigation scenario offers limited health benefits in reducing O_3_‐associated premature deaths. Our projections indicate an increase in premature deaths under the Baseline scenario due to rising VOC emissions. On the other hand, the increases in VOC emissions are suppressed under the Mitigation scenario, leading to small health benefits. The result implies that concurrent emission reduction of both NO_x_ and VOC is crucial for addressing O_3_‐associated premature deaths in South Korea
5. PM_2.5_‐ and O_3_‐associated health burdens in South Korea can potentially benefit from foreign emission reductions. The range of foreign emissions captured in the nine SSP/RCP‐based scenarios maps out a wide spectrum of future possibilities for PM_2.5_‐ and O_3_‐associated premature deaths. This highlights the need for international collaboration in emission reduction to mitigate public health burdens.

## Conflict of Interest

The authors declare no conflicts of interest relevant to this study.

## Supporting information

Supporting Information S1

## Data Availability

The UNEP emission scenarios are detailed in UNEP (2023) available at the UN Environment Document Repository (https://wedocs.unep.org/20.500.11822/42432). The nine SSP/RCP‐based emission scenarios (Riahi et al., 2017) can be assessed through the SSP Public Database–Version 2.0 (https://tntcat.iiasa.ac.at/SspDb) (IIASA, 2018), which is provided by the International Institute for Applied Systems Analysis (IIASA). Estimates of current and future health burdens derived from this study are available at the CU Scholar (https://scholar.colorado.edu/concern/datasets/4m90dx16v) (Choi et al., 2024).
