# Supplementary material for: Source Attribution of Health Burdens From Ambient PM2.5, O3, and NO2 Exposure for Assessment of South Korean National Emission Control Scenarios by 2050
Source: Geohealth. 2024 Aug 3;8(8):e2024GH001042. doi: 10.1029/2024GH001042 (PMC11297529; doi:10.1029/2024GH001042)
Supplement: Supplementary file 1 — Supporting Information S1 [file GH2-8-e2024GH001042-s001.pdf]

GEOHEALTH

Supporting Information for

Source attribution of health burdens from ambient $PM_{2.5}$, O_3, and NO_2 exposure for assessment of South Korean national emission control scenarios by 2050

Jinkyul Choi[1], Daven K. Henze[2], M. Omar Nawaz[3], Christopher S. Malley[4]

[1]Environmental Engineering Program, University of Colorado, Boulder, CO, USA

[2]Department of Mechanical Engineering, University of Colorado, Boulder, CO, USA

[3]Environmental and Occupational Health Department, Milken Institute School of Public Health, George Washington University, Washington, DC, USA

[4]Stockholm Environment Institute, Environment Department, University of York, York, UK

Contents of this file

Text S1
Figure S1
Tables S1 to S8

Introduction

In this supporting information we present a method to estimate 2 m O_3 concentrations in Text S1 (Zhang et al., 2012), South Korea population forecast in 2050 by Statistic Korea in Fig. S1, detailed descriptions of the UNEP emission scenarios in Table S1-S2, a list of SSP/RCP-based scenarios in Table S3, and our estimated future health burdens in Table S4-S8. Table S1 and Table S2 detail the policies and measures implemented in the UNEP Baseline and the Mitigation scenarios, respectively. Both tables are based on information found in Table 3.14-15 of UNEP (2023). Table S3 lists nine SSP/RCP-based scenarios utilized for future emissions outside of South Korea. Table S4 includes our estimated future health burdens using domestic emission scenarios discussed and shown in Sect. 3.4 and Fig. 4 A-D in the main text. Table S5-S8 include our future projection of $PM_{2.5}$-associated premature death, O_3-associated premature death, NO_2-associated premature death, and NO_2-associated childhood asthma incidences, respectively, using domestic and foreign emission scenarios shown in Fig. 4 E-H in the main text. All the future projections are based on our estimated health burdens and emission contributions for the base year 2016.

Text S1

The simulated O3 concentrations at 2 m, $\mathrm{C(2m)}$, is related to the model lowest level concentrations, $\mathrm{C(z_1)}$, following Zhang et al. (2012) as:

$$\mathrm{C(2m)} = \left(1 = \mathrm{R_a(z_1, 2m) v_d(z_1)}\right)\mathrm{C(z_1)}, \qquad \text{Eq. S1}$$

where $\mathrm{v_d(z_1)}$ is O_3 the dry deposition velocity at $\mathrm{z_1}$ and is $\mathrm{R_a(z_1, 2m)}$ the aerodynamic resistance to turbulent transfer from $\mathrm{z_1}$ to 2 m.

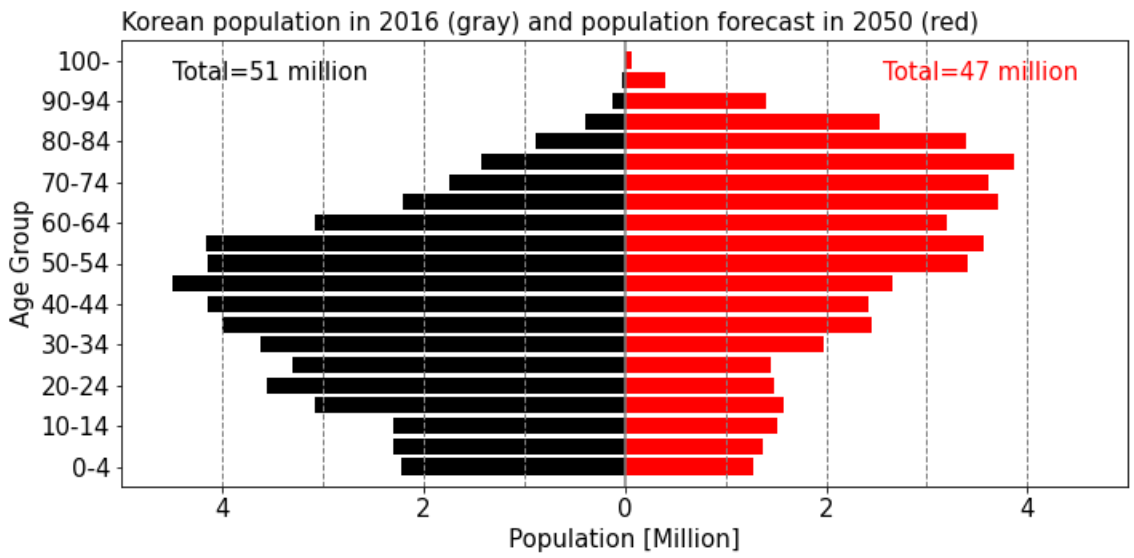

Figure S1. South Korea population in 2016 (black) and population forecast in 2050 (red). Population forecast is provided by Statistics Korea (https://kostat.go.kr/anse/). The total population for each year is shown on the panel.

Table S1. Policies and measures included in the Baseline scenario of UNEP (2023)

**Baseline scenario**		
**Name of plan or policy**	**Name of policy or measure**	**Description**
Status quo	Energy efficiency in industry	
	Energy efficiency in buildings	
	Vehicle emissions standards	
	Energy efficiency	Railroad
	Waste reduction	Recycle, etc
	Electromobility	Road transport
2nd Basic plan for air quality management in Seoul Metropolitan Area (2015-2024)	Tier 4 emissions standards	Off road machinery

Table S2. Policies and measures included in the Mitigation scenario of UNEP (2023)

**Mitigation scenario**		
**Plan or policy**	**Name of policy or measure**	**Description**
2050 Carbon neutrality plan	Transformation sector energy transition	Fuel substitution (from fossil fuels to renewable sources) for electricity generation<br>Fuel substitution for heat generation
	Industrial sector energy efficiency improvement	Energy efficiency improvement<br>Fuel substitution for all industries
	Fuel economy improvement (in road transport)	A fall in the amount of fuel used per kilometer travelled<br>Increase share of small cars
	Transport demand management	Switching from passenger cars to public transport
	Other transport measures	Energy efficiency improvement<br>Fuel substitution
	Fuel substitution	Industrial processes and product use (iron and steel)
2050 Carbon neutrality plan & Urban access regulation (Seoul)	Zero emission vehicle deployment	Full zero-emissions vehicle deployment by 2050 for road vehicle fleet
Carbon neutrality plan for agriculture	Rice cultivation	Modification of flooding pattern
	Livestock management	Livestock nutrition management
Zero energy buildings programme & Building emissions cap programme (Seoul)	Zero energy buildings	Energy efficiency improvement
Municipal waste landfill ban programme	Direct landfill ban	
2nd Basic plan for air quality management in Seoul metropolitan area (2015-2024)	Industrial facility emissions cap and trade programme	Increasing emissions control rates
	Transport demand management	Low emission zone in atmospheric control area in Seoul metropolitan area
	Non-road tier 4 emissions standard	Non-road construction and agricultural machinery
	Improvement of fuel	Decreasing the sulphur content

Table S3. Nine SSP/RCP-based emission scenarios used to estimate ranges of foreign emission impacts on future health burdens attributable to ambient air pollution exposure in South Korea by 2050

Integrated Assessment Model	SSP/RCP Scenarios	Reference
IMAGE	SSP1/RCP1.9<br>SSP1/RCP2.6	van Vuuren et al. (2017)
MESSAGE-GLOBIOM	SSP2/RCP4.5	Fricko et al. (2017)
AIM/CGE	SSP3/RCP7.0<br>SSP3/LowNTCF	Fujimori et al. (2017)
GCAM4	SSP4/RCP3.4<br>SSP4/RCP6.0	Calvin et al. (2017)
REMIND-MAGPIE	SSP5/RCP3.4-OS<br>SSP5/RCP8.5	Kriegler et al. (2017)

Table S4. Estimated future health burdens attributable to air pollution exposure by 2050 using domestic emission scenarios; Fixed emissions of 2016 and the UNEP Baseline and Mitigation scenarios (UNEP, 2023). Population forecasts from Statistics Korea (https://kostat.go.kr/anse/) are applied to all three scenarios. All the future projections are based on our estimated health burdens and adjoint-based emission contributions for the base year 2016.

**Impacts of Domestic Emission Controls on Future Health Burden in South Korea**					
**Pollutant-Burden**	**Domestic Scenario**	**2016**	**2030**	**2040**	**2050**
$PM_{2.5}$-Death	Fixed Emissions	18855	34122	48691	59889
	Baseline		33581	49026	61818
	Mitigation		31670	44076	53982
O_3-Death	Fixed Emissions	3257	6469	10091	13285
	Baseline		6619	10528	13975
	Mitigation		6252	9541	12362
NO_2-Death	Fixed Emissions	8456	15696	22860	28572
	Baseline		10101	13089	15858
	Mitigation		7993	6550	4836
NO_2-Asthma	Fixed Emissions	23194	14719	15052	14162
	Baseline		9389	8493	7741
	Mitigation		7131	3673	1631

Table S5. Estimated future premature death attributable to $PM_{2.5}$ exposure by 2050 using domestic and foreign emission scenarios. Population forecasts from Statistics Korea (https://kostat.go.kr/anse/) are applied to all projections. Domestic emission scenarios are from the UNEP Baseline and Mitigation scenarios (UNEP, 2023). Foreign emission scenarios are from nine SSP/RCP emission scenarios (Riahi et al., 2017); IMAGE SSP1-RCP1.9 and RCP2.6 (van Vuuren et al., 2017), MESSAGE-GLOBIOM SSP2-RCP4.5 (Fricko et al., 2017), AIM/CGE SSP3-RCP7.0 and LowNTCF (Fujimori et al., 2017), GCAM4 SSP4-RCP3.4 and RCP6.0 (Calvin et al., 2017), and REMIND-MAGPIE SSP5-RCP3.4-OS and RCP8.5 (Kriegler et al., 2017). All the future projections are based on our estimated health burden (18,855) and adjoint-based emission contributions for the base year 2016.

**Impacts of Domestic & Foreign Emission Controls on Future $PM_{2.5}$-associated Premature Death in South Korea**				
**Domestic Scenario**	**Foreign Scenario**	**2030**	**2040**	**2050**
Baseline	SSP1-19	28006	39171	48136
	SSP1-26	29320	41221	50630
	SSP2-45	33619	48143	58745
	SSP3-70 (Baseline)	36572	54722	69324
	SSP3-LowNTCF	34439	48629	58038
	SSP4-34	34990	51188	62891
	SSP4-60	35121	51840	64274
	SSP5-34-OS	33945	49339	55960
	SSP5-85 (Baseline)	33945	49339	59452
Mitigation	SSP1-19	26095	34222	40299
	SSP1-26	27408	36271	42793
	SSP2-45	31708	43194	50909
	SSP3-70 (Baseline)	34661	49773	61488
	SSP3-LowNTCF	32527	43679	50201
	SSP4-34	33079	46238	55055
	SSP4-60	33210	46890	56437
	SSP5-34-OS	32033	44389	48124
	SSP5-85 (Baseline)	32033	44389	51615

Table S6. Same as Table S5 but for premature death attributable to O_3 exposure. The health burden for the base year 2016 is 3,257.

**Impacts of Domestic & Foreign Emission Controls on Future O_3-associated Premature Death in South Korea**				
**Domestic Scenario**	**Foreign Scenario**	**2030**	**2040**	**2050**
Baseline	SSP1-19	5036	7727	9855
	SSP1-26	5301	8164	10502
	SSP2-45	6539	10185	13117
	SSP3-70 (Baseline)	7156	11628	15553
	SSP3-LowNTCF	6719	10275	12787
	SSP4-34	6964	11223	14557
	SSP4-60	6823	10904	14051
	SSP5-34-OS	6446	10089	12001
	SSP5-85 (Baseline)	6446	10089	12816
Mitigation	SSP1-19	4669	6741	8242
	SSP1-26	4933	7177	8889
	SSP2-45	6172	9199	11504
	SSP3-70 (Baseline)	6789	10642	13940
	SSP3-LowNTCF	6352	9288	11174
	SSP4-34	6597	10236	12944
	SSP4-60	6456	9917	12438
	SSP5-34-OS	6078	9102	10388
	SSP5-85 (Baseline)	6078	9102	11203

Table S7. Same as Table S5 but for premature death attributable to NO_2 exposure. The health burden for the base year 2016 is 8,456.

**Impacts of Domestic & Foreign Emission Controls on Future NO_2-associated Premature Death in South Korea**				
**Domestic Scenario**	**Foreign Scenario**	**2030**	**2040**	**2050**
Baseline	SSP1-19	9085	11058	12985
	SSP1-26	9449	11784	13845
	SSP2-45	10066	12670	14856
	SSP3-70 (Baseline)	10689	14208	17263
	SSP3-LowNTCF	10166	12773	14638
	SSP4-34	10114	12689	14718
	SSP4-60	10373	13433	15950
	SSP5-34-OS	10471	13786	15137
	SSP5-85 (Baseline)	10471	13786	16198
Mitigation	SSP1-19	6977	4520	1964
	SSP1-26	7341	5245	2824
	SSP2-45	7958	6132	3835
	SSP3-70 (Baseline)	8581	7670	6241
	SSP3-LowNTCF	8058	6235	3617
	SSP4-34	8006	6150	3696
	SSP4-60	8264	6894	4929
	SSP5-34-OS	8363	7247	4116
	SSP5-85 (Baseline)	8363	7247	5177

Table S8. Same as Table S5 but for childhood asthma incidences attributable to NO_2 exposure. The health burden for the base year 2016 is 23,194.

**Impacts of Domestic & Foreign Emission Controls on Future NO_2-associated Childhood Asthma Incidences in South Korea**				
**Domestic Scenario**	**Foreign Scenario**	**2030**	**2040**	**2050**
Baseline	SSP1-19	8448	7176	6340
	SSP1-26	8783	7643	6756
	SSP2-45	9355	8221	7252
	SSP3-70 (Baseline)	9927	9213	8422
	SSP3-LowNTCF	9448	8287	7146
	SSP4-34	9400	8236	7189
	SSP4-60	9636	8713	7784
	SSP5-34-OS	9721	8933	7381
	SSP5-85 (Baseline)	9721	8933	7896
Mitigation	SSP1-19	6190	2356	230
	SSP1-26	6525	2823	646
	SSP2-45	7097	3401	1143
	SSP3-70 (Baseline)	7670	4393	2312
	SSP3-LowNTCF	7190	3467	1036
	SSP4-34	7143	3416	1079
	SSP4-60	7379	3893	1674
	SSP5-34-OS	7463	4113	1272
	SSP5-85 (Baseline)	7463	4113	1787
